# Remarks on the Influence of Mental Cultivation and Mental Excitement upon Health

**Published:** 1845-04

**Authors:** 


### REVIEWS.

---

Art. III.—*Remarks on the influence of Mental Cultivation and Mental Excitement upon Health.* By Amariah Brigham, M.D., Superintendent and Physician of the State Lunatic Asylum, Utica, N. Y. Third Edition. Philadelphia: Lea & Blanchard. 1845. 12mo. pp. 204.

Although Dr. Brigham's work has reached its third edition, and has been republished in Glasgow, Edinburgh, and London, we are not aware that it has ever been presented in a formal manner to the readers of the Journal. As it is intended for general circulation, addressing itself more particularly, however, to parents and to those having charge of the education of children, it of course contains many things which are both common and familiar to the profession, but which, we may be allowed to say, cannot become either too common or too familiar. All of our medical readers may not be interested in it as *parents;* but still, as guardians of the public well-being, as men whose duty and province it is to look after the health, not of the body politic, but the body social, they are or should be interested in it. The views which the book contains are so just and important, and seem to us to have so close a relation both to the duty and business of professional men, that we think it well worth the labor of making a few extracts.

The edition published in Glasgow was edited by Dr. Macnish, who added a preface and some notes; these are incorporated in the present edition, together with the preface of the Edinburgh editor.

We shall pass by the first section, which is wholly occupied with the proposition that the brain is the material organ of the mind. This is a truth which at this day few competent to decide will deny, and one at least which most medical men will admit. The second section refers to the condition of the brain in early life, the effect on the mind of its excitement and enlargement by disease, and to the fact that mental precocity is usually a symptom of disease.

"The brain of a new-born infant weighs about ten ounces; that of an adult, generally, three pounds and a half, apothecaries' weight, frequently a little less. But if the mind of an adult has been long devoted to thought, if he has been engaged in constant study, his brain is usually increased beyond this weight. The brain of Byron, for instance, is said to have weighed four pounds and a half; and that of the illustrious Cuvier, four pounds thirteen ounces and a half.* The size of this organ increases from the time of birth till manhood, remains stationary from this period until old age, and then diminishes in bulk and weight. The relative size of its different portions constantly varies during several of the first years of life, and it is not until about the seventh year that all its parts are formed. During childhood it is "very soft and even almost liquid under the finger, and its different parts cannot be clearly distinguished." Still at this time it is supplied with more blood, in proportion to its size, than at any subsequent period. It then grows most rapidly, and more rapidly than any other organ: its weight is nearly doubled at the end of the first six months; and hence the nervous system, being connected with the brain, is nearly developed, and becomes the predominating system, in youth. At this period of life, however, which is devoted to the increase of the body, it is necessary that the nervous system should predominate; for this system is the source of all vital movement,

---

* There is an error here. Cuvier's brain weighed but 63 ounces. Dupuytren's weighed 64. That of the late Dr. Abercrombie weighed 63. According to Reid, the average weight of the encephalon in a man between 50 and 60 years of age is 50 ounces 2 drachms. See *British and Foreign Medical Review*, for July, 1844. C.

and presides over, and gives energy to those actions which tend to the growth of the organization. Besides, 'infancy,' says Bichat, 'is the age of sensation. As every thing is new to the infant, every thing attracts its eyes, nostrils, &c. That which to us is an object of indifference, is to it a source of pleasure. It was then necessary that the nervous cerebral system should be adapted by its early devolpment to the degree of action which it is then to have.'

"But this great and early development, though necessary for the above purposes, very much increases the liability to disease; it gives a tendency to convulsions, and to inflammation and dropsy of the brain, and to other diseases of the nervous system, which are most common and fatal in childhood.

"It is, therefore, deeply important, that the natural action of the nervous system should not be much increased, either by too much exercise of the mind, or by too strong excitement of the feelings, lest at the same time the liability of children to nervous diseases be increased, and such a predominance given to this system as to make it always easily excited, and disposed to sympathize with disorder in any part of the body."

Dr. Brigham alludes to a number of facts, and adduces several curious cases, showing the effect on the mind of diseased excitement and enlargement of the brain. The intelligent reader can doubtless call to mind many cases of this sort; as also of the kind next noticed, arising from the action on the brain of intoxicating drinks. Unfortunately there are but too many instances that warrant the paradoxical assertion of a man's being most sober when he is most drunk. "Who," says the author, "has not felt that transient joy, happiness, and courage is a marketable commodity, that 'ecstacies can be corked up in bottles, or peace of mind sent in gallons by the mail-coach?"' We subjoin one of the most remarkable examples of precocity and enlargement of the brain quoted by the author; it was furnished to the American Journal of the Medical Sciences, for 1836, by Prof. Horner:

"Master William M., the fourth child of his parents, was born in Philadelphia on the 4th of June, 1820. At birth, his head was of ordinary size, but very soon, after an attack of dropsy of the brain, it began to grow inordinately. After he began to walk, its size was so great that he attracted much attention; and he was apt to fall, especially forwards, from readily losing his equilibrium. His health was generally good.

"Dec. 12th, 1828, he fell against a door, and bruised his forehead; in an hour afterwards he vomited, became very sick, and died the next evening. During his short sickness he had no headache, and complained only of his stomach.

"On examining his head, the day after his death, it was found to be considerably larger than that of a full-grown person, measuring twenty-eight inches in circumference. The lateral ventricles contained a great quantity of transparent serum, which had distended the brain to a very great degree, and produced much of the enlargement of the head. The appearance of all the parts of the brain it is not necessary to particularize. Many parts, especially those at the base of the brain, were healthy, and the small blood-vessels were generally congested with blood.

"The following interesting account of this child's mental and moral faculties, was furnished by Dr. J. K. Mitchell, the family physician: 'When 15 months old, the child spoke well, and, at 18 months, was able to sing a variety of musical airs with tolerable correctness, and always exhibited a strong predilection for music. His intellectual faculties generally were very respectable, and his powers of observation rather remarkable. But his memory, both of language and sentiments, was such as to excite surprise in those who took pains to converse with him. The following example of his powers of recollection may not be amiss. A customer of his father having been absent two years, returned, and, on his entrance into the shop, saluted as an acquaintance, its inmates; but they had forgotten him. On turning to little M., the latter immediately called him by name, inquired kindly about him, and then told him that he had not been to see them for two years.

"'Of a grave and quiet temperament, he preferred the society of his seniors, and took little interest in the common pastimes of childhood. Only sedate children were agreeable to him. For so youthful a person, his sentiments and affections were of a lofty character. Seeing the distress of his mother when commercial affairs took his father to Europe, the child, then five years of age, said, 'Father will soon be back: if he don't come again, I will be a husband to my mother, and will work for her, and take care of her when she is old.'

"For two years before his death, little M. became affected by religious impressions, which grew stronger and stronger, until his death. Often advising others, he presented in his own conduct, a fine exemplification of his principles, being distinguished among the children of the family and the school, for love of truth, and general sincerity of character. At length, even while in full health and vigor, he spoke of death as a thing to be desired; and when dying, expressed pleasure at the approaching crisis."

Dr. Brigham illustrates this branch of his subject by a reference to the enlarged brain and early mental development of rickety children. He adverts also to the danger of mental excitement in children of scrofulous diathesis, in whom there is ofttimes a rare development of mind. This danger is rendered more striking by a fact which the author does not notice. Recent pathological investigations have shown the extreme liability of strumous children, from 2 to 10 years of age, to *tubercular meningitis.* It is more insidious in its approach even than the ordinary form of meningitis, and once established nearly always pursues its march uncontrolled. Hence the increased necessity of studiously avoiding every thing that will unduly excite the mind, of keeping in check the early maturity of understanding when it exists. Safety is only to be found in aiding by all possible means the development and improvement of the other organs of the body.

The third section is devoted to a consideration of the consequences which have resulted from inattention to the connexion between the body and mind, and to the illustration of the fact that the best minds are not produced by early mental culture. There is truth we think in the following paragraph:

"Teachers of youth, in general, appear to think, that in exciting the mind, they are exercising something totally independent of the body,—some mysterious entity, whose operations do not require any corporeal assistance. They endeavor to accelerate, to the utmost, the movements of an extremely delicate machine, while most unfortunately they are totally ignorant or regardless of its dependence on the body

They know that its action and power may both be increased for a while, by the application of a certain force; and when the action becomes deranged, and the power destroyed, they know not what is the difficulty, nor how it can be remedied. Fortunately they do not attempt to remedy it themselves, but call in the physician, who, if he affords any relief at all, does it by operating on a material organ. If medical men entertained the same views as teachers, they would, in attempting to restore a deranged mind, entirely overlook the agency of the body, and instead of using means calculated to effect a change of action in the brain, would rely solely upon arguments and appeals to the understanding. For if the mind may be cultivated independent of the body, why may not its disorders be removed without reference to the body?"

The author inveighs strongly and justly against the excessive abundance of *books for children*, and the absurdity, nonsense, and falsehood, contained in many of them.

"Where is the proof that they have ever benefited a single child? Do the youth now, of the age of 15, who have used such books the most of their lives, who committed to memory innumerable truths, and were taught to reason when at the age of 3 or 4, possess more active and independent minds than their parents possessed at the same age? Does their mental power *now* show the *good* effect of their early and extraordinary culture? Do not the numerous slender, delicate and pale-faced youths who are seen in our colleges, and in boarding schools for girls, exhibit the *bad* effects of this system? I ask again, where is *any* evidence that books, put into the hands of children before the age of seven or eight, are of any lasting benefit, either to the body or the mind?"

The mode of teaching also comes in for a share of censure:

"The method of teaching little children varies in different schools; but that is everywhere considered the *best* which forces the infant mind the *fastest.* In some schools the *memory* is chiefly cultivated, and children are taught innumerable facts. Here we see those who are scarcely able to talk exhibited as wonderful children. They are declared to be deserving of the highest praise, and prophesied about as giving promise of great distinction in future, because they are able to tell us who was the oldest man, and many other equally useful and important facts. They are also able to tell us many truths in astronomy, geometry, chemistry, &c. &c., of which the innocent beings know about as much as do parrots of the jargon they deliver. In other schools teachers are opposed to such practice; and say that a child should learn nothing but what he understands; that the memory should not alone be cultivated; therefore they teach children that Methuselah was not only the oldest man, and nine hundred and sixty-nine years of age, but that he was the son of Enoch, and the grandfather of Noah, and that a year means three hundred and sixty-five days, and a day twenty-four hours; and all this they teach in order, as they say, that a child may *fully understand* what he learns. Other teachers say that it is very wrong to *compel* a child to learn—very wrong indeed; and that he should learn no more than he will cheerfully; but though they do not gain their purpose by exciting *fear*, they awaken other passions of the strongest kind in the child, by a system of *rewards* and of *praise.*"

Dr. Brigham professes to speak on this subject from personal observation. The observation of others can supply the parallel to what we have just quoted, and also to the following:

"I have seen a course like the following pursued in many families in various parts of the country, and I know that this course is approved of by many excellent persons. Children of both sexes are required, or induced, to commit to memory many verses, texts of scripture, stories, &c., before they are three years of age. They commence attending school, for six hours each day, before the age of four, and often before the age of three; where they are instructed during three years in reading, geography, astronomy, history, arithmetic, geometry, chemistry, botany, natural history, &c. &c. They also commit to memory, while at school, many hymns, portions of the Scriptures, catechisms, &c. During the same period they attend every Sunday and Sabbath school, and there recite long lessons; some are required to attend upon divine service at the church twice each Sunday, and to give some account of the sermon. In addition to these labors, many children have numerous books, journals, or magazines to read, which are designed for youth. I have known some required to give strict attention to the chapter read in the family in the morning, and to give an account of it; and have been astonished and *alarmed* at the wonderful power of memory exhibited on such occasions by children when but five or six years of age. I have known other children, in addition to most of the above performances, induced to learn additional hymns, chapters of Scripture, or to read certain books, by the promise of presents from their parents or friends."

As consequences of a course like this, the author says he has seen children die at the age of six or seven years, exhibiting to the last a maturity of understanding that only increased the agony of a separation; others grow up to manhood with feeble bodies and disordered nervous systems; others, and perhaps the most numerous class, exhibit in after life a small degree of intellectual power, and become the passive instruments of those who in early years were accounted their inferiors.

The observations contained in our next extract are unquestionably true. One memorable and immortal instance will suggest itself to the medical reader—we need scarcely name John Hunter. Besides the examples among our countrymen adduced by the author, others are not wanting both of the past and present. Patrick Henry's early culture consisted mainly of trout-fishing; Hamilton, than whom few more remarkable men have ever lived in any country, was a merchant's clerk; Channing, beloved during life, and whose fame is growing brighter and brighter every day, spent his youth wandering among the green fields and forests; Sparks pushed the jack-plane even after he had arrived at manhood; Cooper was a naval officer.

"The history of the most distinguished men will, I believe, lead us to the conclusion, that early mental culture is not necessary in order to produce the highest powers of mind. There is scarcely an instance of a great man, one who has *accomplished* great results, and has obtained the gratitude of mankind, who in early life received an education in reference to the wonderful labors which he afterwards performed. The greatest philosophers, warriors, and poets, those men who have stamped their own characters upon the age in which they lived, or who, as Cousin says, have been the 'true representatives of the spirit and ideas of their time," have received no better education, when young, than their associates who were never known beyond their own neighborhood. In general their education was but small in early life. *Self-education*, in after life, made them great, so far as education had any effect. For their elevation they were indebted to no early *hot-house culture*, but, like the towering oak, they grew up amid the storm and the tempest raging around. Parents, nurses, and early acquaintances, to be sure, relate many anecdotes of the childhood of distinguished men, and they are published and credited. But when the truth is known, it is ascertained that many, like Sir Isaac Newton, who, according to his own statement, was 'inattentive to study, and ranked very low in the school until the age of twelve;" or, like Napoleon, who is described, by those who knew him intimately when a child, as 'having *good health*, and in other respects was like other boys,'† do not owe their greatness to any early mental application or discipline. On the contrary, it often appears that those who are kept from school by ill health or some other cause in early life, and left to follow their own inclination as respects study, manifest in after life powers of mind which make them the admiration of the world."*

As a practical commentary on what is here stated, Dr. Brigham gives the following sketch of one of the most profound and distinguished jurists of this country.

"'I was brought up among the highlands and hilly parts of Connecticut, and was never kept on the *high-pressure* plan of instruction. It was not then the fashion. I went to school, and studied in the easy, careless way, until I went to college. I was daily, and sometimes for a month or more, engaged in juvenile play, and occasional efforts on the farm. I was roaming over the fields, and fishing, and sailing, and swimming, and riding, and playing ball, so as not to be but *very superficially learned*, when I entered college. I was not in college half the time. I was at home, at leisure, or at gentle work, and much on horseback, but never in the least dissipated. I easily kept pace with my class, for it was in the midst

* Shakspeare, Moliere, Gibbon, T. Scott, Niebuhr, W. Scott, Byron, *Franklin*, *Rittenhouse*, *R. Sherman*, Professor Lee, Gifford, Herder, Davy, Adam Clarke, &c.

† Memoirs of the Duchess of Abrantes.

of the American war, and there no scholars, or much stimulus to learn. Silent leges inter arma. When I went to study law, I had my own leisure, and great exercise and relaxation in enchanting rides, and home visits, until I got to the bar. I lived plain, drank nothing but water, ate heartily of all plain wholesome food that came in my way; was delighted with rural scenery, and active and healthy as I could be. Here I laid the *basis of a sound constitution*, in which my brain had not been unduly pressed or excited, and only kept its symmetry with the rest of the animal system. It was not until I was twenty-four that I found I was very superficially taught, and then *voluntarily betook myself to books*, and to learn the classics, and everything else I could read. The ardor and rapidity with which I pursued my law and literary course, was great and delightful, and my *health and spirits* were sound and uniform, and neither has faltered, down to this day.'"*

The early history of Walter Scott, quoted in another place, is to the same purport. He, however, at last died of softening of the brain, induced by vast mental labor in the latter years of his life.

To go back a little, may we not say with the author, that "it is a great mistake to suppose children acquire no knowledge while engaged in voluntary play and amusements."

"They thus do acquire knowledge as important as is ever acquired at school, and acquire it with equal rapidity. Many think that the child who has spent the day in constructing his little dam, and his mill, in the brook, or the stream that runs in the gutter; or in rearing his house of clods or of snow, or in making himself a sled or cart, has been but idle, and deserves censure for a waste of his time, and a failure to learn anything. But this is a great error of judgment; for, while he has thus followed the dictates of nature, both his mind and body have been active, and thereby improved. To him anything which he sees and hears and feels is new, and nature teaches him to examine the causes of his various sensations, and of the phenomena which he witnesses. For him, the Book of Nature is the *best book*, and if he is permitted to go forth among the wonders of creation, he will gather instruction by the eye, the ear, and by all his senses.

"He is for a while just as ignorant that stones are hard, that snow will melt, that ice is cold, that a fall from the tree

* Chancellor Kent. This was written after reading a copy of the first edition in 1833; and was not intended for publication.

will hurt him, and a thousand other common facts, as he is of a 'parallelogram,' or 'perimeter,' or the 'diameter of the sun,' or the 'pericarpium of flowers,' or of many other similar things, which some think important for infants to know. If his time is constantly occupied in learning the last, he will grow up ignorant of many common truths, and fail in the best of all learning, *common sense.*

"The child, when left to himself, manifests a true philosophical spirit of inquiry. The story related of the celebrated Schiller, who, when a boy, was found in a tree, during a thunder-storm, trying to find where the thunder and the lightning came from, is an instance of the natural tendency of every child to self-education. This tendency it is highly important to encourage, for it involves the cultivation of that spirit of inquiry 'which is far more valuable than limited acquirements in knowledge; a spirit which teaches us to distinguish what is just in itself, from what is merely accredited by illustrious names; to adopt a truth which no one has sanctioned, and to reject an error of which all approve, with the same calmness as if no judgment was opposed to our own.' But this spirit will never be acquired, when the child is taught from his infancy to depend upon others for all he knows, to learn all he does learn as a task, and not from the desire of ascertaining the truth and gratifying his curiosity.

"Let not the parent, therefore, regret that his child has passed his early hours out of school; for in all probability the knowledge he has gained while running and exercising in the open air at play, is more valuable than any he would have gained at school. At all events, he has gained what is far, very far more valuable than any mental acquirements which a child may make, viz: a sound body, well-developed organs, senses that have all been perfected by exercise, and stamina which will enable him in future life to study or labor with energy and without injury."

In the fourth section the author fortifies himself with the opinion of celebrated physicians respecting early mental cultivation.

In the fifth he treats of the influence of mental cultivation and mental excitement in producing insanity, nervous affections, and diseases of the heart. In reference to insanity, the author's position now, and for a number of years past, at the head of a lunatic asylum, entitles his opinions to more than ordinary weight. After glancing at certain facts in reference to this disease, he mentions the following as among the causes of its great prevalence in this country:

"First. Too constant and too powerful excitement of the mind, which the strife for wealth, office, political distinction, and party success, produces in this free country.

"Second. The predominance given to the nervous system, by too early cultivating the mind and exciting the feelings of children.

"Third. Neglect of physical education, or the equal and proper development of all the organs of the body.

"Fourth. The general and powerful excitement of the female mind."

The first two causes may be illustrated by certain facts relative to the city of Hartford (the residence of the author at the date of the first edition, 1832), "which are probably applicable to many if not most of the towns of the same size in the United States."

"This city contains about 7,000 inhabitants. Nearly all, if not all, the children of the city, commence attending school as early as the age of three or four, and attend six hours each day, for several years. Nearly all attend school on the Sabbath also. Most families have a library and books for children, besides newspapers and other periodicals. There are nine large churches in the city, belonging to six different denominations, exclusive of one for colored people. These are all well filled twice, and frequently three times, every Sunday. Besides, there are religious meetings on other days, amounting, in the various churches, to twenty or thirty during the week. There are two lyceums, or literary associations, both of which meet once a week, and are open to all, without expense. At one, are weekly debates, usually on some political or historical subject; and at the other is a lecture every week, on such subject as the lecturer pleases. Both of these are well attended. Every week, *seven* large political newspapers, advocating the interests of three different parties, are published in Hartford; and also *five* large religious newspapers; no two of which belong to the same sect. Several other periodicals are published here, but not weekly. In addition to the papers published in this town, men of business take one or more of those published in the larger cities, and most of the reviews and magazines of this country, and of England, are received here and read."*

The evils resulting to both sexes from the neglect of physical education, are acknowledged by all; and the subject has attracted the attention of some of the best minds in the profession both at home and abroad. The ancients understood its value far better than we moderns—we who boast of our vast superiority in knowledge. With them the education of the body, if the expression may be allowed, was of paramount importance. Their gymnasia, their games, in which ofttimes sturdy manhood contended with supple and pliant youth, their martial sports and exercises, all combined to give them what we know they had, the soundest minds in the *soundest bodies*, the latter presenting the noblest specimens of physical conformation that the world has ever beheld. "Charmed in the Grecian stone," there they still stand, after the lapse of two thousand years, the wonder and the delight of mankind; and there too, according to a recent traveler, is still to be seen, in the descendants of that noble old race, "the one glorious shape transcendant in its beauty."

We hurry over the remainder of this section, which is occupied with organic diseases of the heart as a result of mental excitement. The author thinks them fearfully on the increase. We cannot forbear quoting a couple of sentences from a note furnished by Dr. Macnish.

"It is very common for young medical students to imagine they labor under aneurism of the aorta or some other form of disease connected with the heart or its great outlet, especially if their teachers touch strongly upon this subject. Such is especially the case with hard-working and assiduous youths, whose over-tasked brains induce palpitation of the heart."

---

* On inquiry at the post-office, I learn that eighty daily, one hundred and ten semi-weekly, and four hundred and thirty-two weekly newspapers, published in other places, are taken by the inhabitants of Hartford. Besides more than three hundred dollars are annually received at the same office for postage on papers and pamphlets that are received irregularly.

The experience of all of our readers will tally with that here set forth. We have a pleasant reminiscence of a fellow-student of our own (a most "hard-working" and "assiduous" one he was too) who more than once labored under hypertrophy with dilatation, had at divers times aneurism of the aorta, and survived all sorts of valvular diseases.

Section sixth is occupied with the subject of moral education. We commend the following extracts to our *parental* readers:

"Such effects cannot, however, be produced by *precepts* alone. Children should be induced to *practise* the virtues which it is intended they shall possess, and by *practice* they should be endowed with good propensities and good habits. If parents would but feel that it is as essential to teach a child to practise the virtues that are desirable, as it is to cultivate the mind, and would give as much attention to the early development and proper exercise of the affections and passions, and take that pains to develope all the natural excellences of his character they do to accelerate his progress in knowledge, we should soon see a great and pleasing change in the dispositions and conduct of men. But now, in very many families, the greatest praise is not bestowed upon those children that are merely *good*, but upon those whose minds are most active and premature. In schools, much of the praise and censure, reward and punishment, connected with early mental culture, is calculated to awaken rivalry, envy, and hatred. Moral culture is sacrificed, in early life, to intellectual, and the bad passions are called forth to aid the mind's improvement; and then what originates from a faulty or neglected moral education is considered the fault of nature itself. But nature has not had fair play.

"*Example* is also of great importance in the education of children, in consequence of their natural propensity to imitation. * * * * Parents who are constantly manifesting fretful and unhappy dispositions, will do much towards producing like dispositions in their children. From these observations, those who have the care of educating children, cannot fail to see the importance of the examples they set them; they will also reflect that whatever is inculcated upon children by precept is of trifling consequence, compared with that which they learn by example; and if they wish to have their children possess a spirit of benevolence, kindness, or humility, they must cherish and cultivate these virtues in themselves, and be particularly careful not to let any contradiction exist between their expressed opinions of the value of these dispositions and their own habitual conduct."

In section seventh the author argues that cultivation of the mind at a proper time of life is beneficial to health. He cites Brunaud to show that literary men have in all countries usually been long-lived. The fact is also evident from a table he gives at the end of the volume, showing the ages attained by distinguished personages both of ancient and modern times. The following statement contained in one of the author's notes will interest our readers:

"The Declaration of American Independence was signed by 56 delegates—35 from the Northern, and 21 from the Southern States. But *one* survives, and only *two* have died from accident. The whole number of years lived by these delegates, not including the two mentioned, is 3,609; or 66 years and 9 months each. Those from the Northern States average 70 years and a half, and those from the States at the South, a little less than 60."

In further illustration of this subject he says that the advance of knowledge and civilization has led to scientific discoveries which have saved thousands of lives, to the building of hospitals and charitable institutions by which life has been preserved and prolonged, and to the overthrow of superstitions that formerly destroyed an immense number of human beings. Bills of mortality show that the probability of life is greatest among the most refined and cultivated nations. Intellectual cultivation is beneficial by subjecting the sensual appetites and passions to the control of reason. According to the author it led to the reformation in the use of intoxicating drinks in this country, temperance societies being but the *effect* of the general diffusion of knowledge.

In the eighth and last section, Dr. Brigham discusses the influence of mental cultivation in producing dyspepsia in literary men. He thinks irritation of the brain by far the most frequent cause of this disease. We cannot follow the author, much as we wish to do so, in his illustrations of this branch of his subject, but must here bring our notice to a close. We heartily concur, however, in his recommendation to "those who are worn down by mental labor and anxiety, connected with long-continued disorder of the digestive organs, to throw aside their *bitters*, *blue-pills*, *mustard seed*, *bran bread*, *&c.*, *&c.*, and seek bodily health and future mental vigor, in judicious exertion of the body, innocent amusements, cheerful company, ordinary diet, and *mental* relaxation."

We hope this imperfect notice of the book will help to make it known; and that the views which it inculcates may be deeply pondered on. History, including that of our own day and our own times, is full of mournful examples of men struck down in a brilliant and honorable career; or who "brokenly live on," in bodily and mental imbecility. Unfortunately, precepts and rules, warnings and signs, however strongly urged, and however plainly perceived, are too often totally disregarded—"seeing they will not perceive; hearing they will not understand." Two instances occur to us at this moment of distinguished men, in different spheres of life, who have recently fallen victims to severe mental labor. The one is a Senator from Massachusetts, the Hon. Mr. Bates, a man of great moral and intellectual worth, who died a few days ago at Washington. In the eloquent tribute to him by his colleague, Mr. Webster, it is stated that his fatal illness was brought about by his excessive application to the duties of an important committee of which he was chairman, and to the more general ones appertaining to his senatorial office. His friends saw that he was over-tasked, and urged upon him the necessity of relaxation—but to no purpose. The other is the late editor of the New York Journal of Medicine, Dr. Forry, along whose brief but bright career there went high hopes, not all unfulfilled, and over whose mortal resting-place there gathers a bright and holy light. He too had his warnings, self-perceived, for they came in no questionable shape, and again and again pointed out by his friends; but despite them all, and despite those maxims and precepts which none knew better than himself how to counsel, he persisted in his protracted vigils and toils, even when he could scarcely assume the position necessary to write,

> "Like an inspired and desperate alchemyst,
> Staking his very life on some dark hope."

And thus it is to be feared it will ever be. Unquenchable thirst of knowledge, burning desires, and lofty aspirations—these now as ever will hold their wild sway over the human heart and the human mind; and year after year, youth of promise, manhood of vigor, and old age of greenness and freshness, will be sacrificed to the love of fame, the struggle for power, and the search for glory. C.

